# Examining sound levels across different time scales measured from body-worn dosimeters

**DOI:** 10.1121/10.0035807

**Published:** 2025-02-27

**Authors:** Erik Jorgensen, Jennifer B. Tufts, Erika Skoe

**Affiliations:** 1 Department of Communication Sciences and Disorders, University of Wisconsin-Madison, Madison, Wisconsin 57306, USA; 2 Department of Speech, Language, and Hearing Sciences, University of Connecticut, Storrs, Connecticut 06269, USA

## Abstract

Studies are increasingly investigating listeners' acoustic environments using real-world data collection methods to personalize interventions for hearing loss and understand individual differences in intervention outcomes. A pressing methods question is the extent to which the time scale of the sample and number of sampling periods need to be considered. The purpose of this study was to characterize the extent to which the sound levels in a listener's vicinity, one common measure of acoustic environments, change across different time scales. Listeners wore a personal noise dosimeter continuously for one-week sampling periods at three time points. The effects of season, week, day of the week, and time of day on acoustic environment demand (proportion of samples ≥ 40 dB LAeq and mean sound levels for samples ≥ 40 dB LAeq) and diversity (the distribution of LAeq values, quantified by entropy) were characterized. Acoustic environment demand and diversity were relatively similar across seasons and weeks but varied more between days and across the day. Results suggest that a single one-week sampling period, collected at any time of year but balanced across days of the week and time of day, may capture sufficient information about a listener's acoustic environments to inform decisions about interventions.

## I. INTRODUCTION

Finding effective solutions to real-world listening difficulties requires an understanding of the acoustic environments that listeners experience in their daily lives. Recent years have seen a proliferation of work that aims to characterize such real-world acoustic environments, particularly, for listeners with hearing loss. This body of work has yielded important insights into some characteristics of the acoustic environments that listeners experience and factors that affect those environments. Understanding the typical acoustic environments of listeners and how those environments are affected by factors, such as age, hearing loss, and lifestyle, can help tailor intervention design and improve intervention outcome measurement in daily life. For example, listeners who experience more demanding or diverse acoustic environments may benefit from more sophisticated hearing aid signal processing technologies. However, important questions regarding sampling methods remain. In particular, it is unknown how long of a sampling period and/or how many sampling periods are required to adequately represent a listener's daily acoustic environments. If acoustic environments change significantly across time periods, design and interpretation of studies characterizing real-world acoustic environments must take these temporal effects into account.

There are many terms related to the acoustic environment that are used in different ways by researchers, including auditory ecology (Gatehouse *et al.*, 1999), auditory lifestyle (Cox *et al.*, 2011; Lelic *et al.*, 2022; Wu and Bentler, 2012), listening environment (Klein *et al.*, 2018; Leonard *et al.*, 2013), acoustic environment (Busch *et al.*, 2017; Jorgensen *et al.*, 2023a; Klein *et al.*, 2018), and auditory reality (Noble, 2008). Two terms related to the sound of the environment have standardized definitions—acoustic environment and soundscape (Davies, 2012; ISO, 2014; Schafer, 1977; Southworth, 1969; Wagener *et al.*, 2008)—and recent efforts have aimed to bring consensus around what these terms mean and how they are used (e.g., Mitchell *et al.*, 2024). Acoustic environment is defined by ISO (2014) as “sound at the receiver from all sound sources as modified by the environment.” Soundscape is distinguished from acoustic environment in that soundscape refers to how the acoustic environment is perceived by a listener, including how that perception is affected by context such as information from other sensory modalities, the listeners' sociocultural position, the listening activity, etc. (Grinfeder *et al.*, 2022; Mitchell *et al.*, 2024). In this study, we use acoustic environment because we consider only aspects of sound *received* by the listener rather than *perceived* by the listener. Although acoustic environment is often used to refer to a specific place which may be experienced by different receivers over time, we use it in this study to mean the acoustic environment of the *listener*. That is, we are interested in how the acoustic environment of a specific listener changes over time (and, presumably, place) rather than how the acoustic environment of a place changes and is experienced by different listeners at different times. Acoustic environments form a vitally important component of listeners' cumulative reality and influence many aspects of health and well-being (Noble, 2008; Schafer, 1977; Stansfeld *et al.*, 2000). The types of acoustic environments that listeners experience affect their perceptions of their own hearing ability or disability, communication success, and hearing loss intervention benefit (Gatehouse *et al.*, 2006; Jensen and Nielsen, 2005; Noble, 2008; Skoe *et al.*, 2019; Wu and Bentler, 2012). Systematically characterizing the acoustic environments and factors that affect the types of acoustic environments listeners experience is critical to understanding variance in hearing disability and intervention benefit, as well as designing hearing aid and cochlear implant technologies to improve real-world outcomes. Characterizing listener acoustic environments may also give deeper insight into how the auditory system dynamically adjusts to the prevailing acoustic conditions (Dean *et al.*, 2005; Parker *et al.*, 2022) or how acoustic environments can shape auditory abilities (e.g., Merten *et al.*, 2021; Slater *et al.*, 2015; Worschech *et al.*, 2021).

One major challenge in characterizing listeners' acoustic environments is that it requires collecting granular, contextualized data from the real world (Francis, 2022; Keidser *et al.*, 2020). Collecting data in the real world is logistically challenging, expensive, and time-consuming. The feasibility of this type of research hinges, in part, on how long of a sample is required to be representative of a listener's acoustic environments in general. Knowing either the shortest sample duration or the fewest number of samples that can be considered representative—or at least knowing the effects of under-sampling in time or number of samples—is essential to the planning, undertaking, and interpretation of research on listeners' real-world acoustic environments.

## II. PREVIOUS WORK ON LISTENERS' REAL-WORLD ACOUSTIC ENVIRONMENTS

Most work characterizing listeners' real-world acoustic environments has used one of three data collection methods: audio recordings from body-worn devices (Benítez-Barrera *et al.*, 2020; Benítez-Barrera *et al.*, 2023; Chan *et al.*, 2023; Klein *et al.*, 2018; Jensen and Nielsen, 2005; Smeds *et al.*, 2015; Wagener *et al.*, 2008; Wu *et al.*, 2018), ecological momentary assessments (Edinger *et al.*, 2021; Jensen and Nielsen, 2005; Jorgensen *et al.*, 2021; Walden *et al.*, 2004; Wu *et al.*, 2018), or acoustic metadata taken from devices such as hearing aids, cochlear implants, smartphones, smartwatches, or dosimeters (Busch *et al.*, 2017; Camera *et al.*, 2019; Christensen *et al.*, 2021; Edinger *et al.*, 2021; Flamme *et al.*, 2012; Gatehouse *et al.*, 2006; Humes *et al.*, 2018; Kaf *et al.*, 2022; Mueller *et al.*, 2008; Jorgensen *et al.*, 2023a; Jorgensen *et al.*, 2023b; Parker *et al.*, 2022; Pearsons *et al.*, 1977; Skoe *et al.*, 2019; Tufts and Skoe, 2018; Tufts *et al.*, 2020; Wu and Bentler, 2012). How acoustic environments are characterized depends on the technology used to collect data. One of the most common metrics is sound *levels*, which can be directly recorded by hearing devices and dosimeters and easily calculated from recordings (e.g., Christensen *et al.*, 2021; Humes *et al.*, 2018; Jorgensen *et al.*, 2023a; Pearsons *et al.*, 1977; Skoe *et al.*, 2019; Smeds *et al.*, 2015; Wu *et al.*, 2018). Sound *classification* is also commonly used, as classes are recorded by hearing devices, can be derived from audio recordings, and reported on ecological momentary assessment (e.g., Benítez-Barrera *et al.*, 2020; Busch *et al.*, 2017; Humes *et al.*, 2018; Klein *et al.*, 2018; Mueller *et al.*, 2008; Wu *et al.*, 2018). Data from such studies—even if collected over a relatively long period of a month or more—are typically collapsed across time to characterize average environment characteristics. Studies characterizing sound levels have shown that levels can vary widely across individuals and vary as a function of age, gender, location, culture, occupation, listening activity, and method of estimation (Benítez-Barrera *et al.*, 2023; Chan *et al.*, 2023; Flamme *et al.*, 2012; Jorgensen *et al.*, 2023a; Tufts and Skoe, 2018; Wu and Bentler, 2012). The peakedness of the sound level distribution varies at least as a function of age, with older listeners showing more peaked distributions (Christensen *et al.*, 2021; Jorgensen *et al.*, 2023a; Jorgensen *et al.*, 2023b; Wu and Bentler, 2012). Studies characterizing sound classes generally show that adult listeners spend most of their time either in quiet or listening to speech. On average, the acoustic environments of adult listeners were found to comprise 50%–60% speech (either conversation or media), 30%–40% quiet, 5%–10% music, and 5%–10% noise (for a review, see Wolters *et al.*, 2016). Among all of these studies quantifying acoustic environments, the sampling time ranged from 2 to 425 days. Two of the studies (Busch *et al.*, 2017; Humes *et al.*, 2018) used long sampling periods of 364 days and 425 days, respectively. Excluding those two sampling periods, the average sampling period was 24 days. Data from these studies were collected continuously or nearly continuously rather than at discrete time points. However, the broad tendency in the literature is to boil down samples—be they 2 days or 425 days—to one or two numbers representing the entire sample period, potentially obscuring meaningful within-sample variation. For example, Humes *et al.* (2018), Smeds *et al.* (2015), Wagener *et al.* (2008), and Wu *et al.* (2018) described the sound levels of acoustic environments among adult hearing aid users by employing a variety of different sampling period durations, but they generally grouped the data across many time points, providing average characterizations of acoustic environments across weeks, months, or years.

Five studies did include a temporal analysis of the acoustic environment data collected, however,

• In a brief report, Tufts *et al.* (2020) reported that average sound levels, collected from college-aged listeners who wore dosimeters for three week-long runs, were moderately to robustly correlated across weeks.
• Christensen *et al.* (2021) investigated hearing aid use patterns throughout the day among hearing aid users, finding that hearing aid use across the day fell into one of four clusters—sparse use, use throughout the day, morning use, or evening use. Although they could not fully characterize how the acoustic environment changed across the day due to only having measurements while hearing aids were worn, they did find that acoustic environment demand (average sound levels) and diversity [sound level standard deviation (SD)] increased with increasing hearing aid use time. Treating day of the week as a random effect, they also showed that hearing aid use was lower at the beginning of the week than at the end of the week.
• Humes *et al.* (2018) compared hearing aid data-logging after 6 months of use to data-logging after 1–2 years of use (13 months on average). Characterizing the acoustic environment using sound classes, they found that although the proportion of time spent listening to speech in noise was larger in the 1–2-year data-logging period than the 6-week data-logging period, the change was relatively small (increase of approximately 3%). In general, the acoustic environments experienced remained relatively stable between the two measurements. Again, the results of that study should be interpreted with caution as acoustic environment measurements could only be taken while hearing aids were worn. Further, that study did not specifically look at how the acoustic environment changed on a variety of time scales.
• Tufts and Skoe (2018) investigated noise doses (percentages of a recommended limit of daily exposure, taking into account sound level and exposure time) among a group of musician and nonmusician college students who wore dosimeters continuously for 1 week. They found that musicians experienced higher sound levels than nonmusicians on all days of the week, but there was a day-of-the-week interaction such that the degree of difference was greatest on the days that coincided with marching band rehearsals and performances. It is possible that had the musicians and nonmusicians been sampled at a different time point—for example, outside of marching band season—differences in the two groups may have been smaller. This raises the question of how much *when* acoustic environment measurements are taken matters to the representativeness of the measurement.
• Flamme *et al.* (2012) investigated daily noise exposure levels for 286 adults living near Kalamazoo, MI. Study participants wore dosimeters for several days (median = 9.8 days). As in Tufts and Skoe (2018), Flamme *et al.* (2012) found differences across days of the week. They reported higher median levels on Thursday, Friday, and Saturday than the rest of the week, although the largest difference (Thursday–Sunday) was only about 2 dB. They also examined sound levels across the day, finding that levels were lowest during the 3-h interval ending at 6:00 AM, rising to a peak during the 3-h interval ending at 6:00 PM, and then decreasing.

Taken together, these findings suggest that sampling period—on the day and time-of-day level—could have an impact on the resulting acoustic environment measurements but one might ask whether, in practice, this matters. Our contention is that temporal factors are an underappreciated and undermeasured variable in studies of acoustic environments with questions remaining to be answered: Are sound levels similar from month to month or between the winter and fall? If studies use short sampling periods on the scale of 3–4 days (e.g., Smeds *et al.*, 2015; Wagener *et al.*, 2008), do results differ if the data are collected from Friday to Monday or Monday to Thursday? If they use long sampling periods on the scale of a month or more (e.g., Edinger *et al.*, 2021; Klein *et al.*, 2018; Wu *et al.*, 2018), do results differ from week one to week four? Can sound levels or classes be meaningfully compared among studies employing different sampling windows? Some studies have anecdotally reported that 1 week of sampling is representative of a listener's typical acoustic environments (Schinkel-Bielefeld *et al.*, 2024) but is this actually the case? These are critical questions; if acoustic environments change significantly across time points, research on acoustic environments must take this into account either by collecting data at multiple time points or interpreting data with respect to when it was collected.

The purpose of the present study is to address this gap with an emphasis on how sound levels differ across time scales. However, whether sound levels *qua* sound levels change across different time scales is not principally of interest here. Rather, sound levels, which are easy to estimate using inexpensive wearable devices, are used as a convenient proxy to characterize how *demanding* and *diverse* acoustic environments are (Gatehouse *et al.*, 2003, 2006; Wu and Bentler, 2012; Christensen *et al.*, 2021). Higher sound levels suggest a more demanding acoustic environment: Higher sound levels indicate potentially higher background noise levels, poorer signal-to-noise ratios, more noise sources, and greater acoustic complexity (Christensen *et al.*, 2021; Ghozi *et al.*, 2015; Humes *et al.*, 2018; Jorgensen *et al.*, 2023a; Jorgensen and Wu, 2023; Smeds *et al.*, 2015; Wu and Bentler, 2012; Wu *et al.*, 2018). Sound levels can also be used to characterize acoustic environment diversity by examining the distribution of the levels (Christensen *et al.*, 2021; Jorgensen *et al.*, 2023b; Wu and Bentler, 2012). If a listener's acoustic environments, whether demanding or not, do not change much over time, the distribution of sound levels will be very narrow and, thus, the acoustic environments will be very predictable, i.e., low in diversity. This predictability can be quantified by calculating the entropy of the distribution. Narrow distributions of levels in which the acoustic environments are predictable (i.e., have low diversity) result in low entropy, whereas uniform distributions of levels in which the environments are unpredictable (i.e., have high diversity) result in high entropy (Jost, 2006; Jorgensen *et al.*, 2023b; Sherwin and Prat, 2019; Wu *et al.* 2023). The present study quantified acoustic environment demand and diversity from sound levels collected from noise dosimeters worn by a group of listeners for 1 week at three different time points in the academic year. Using three metrics calculated from dosimeter samples, we aimed to determine whether acoustic environment demand and diversity varied across different seasons, weeks, days of the week, and time of day. In short, we ask the question: Are acoustic environments more demanding or diverse during certain seasons, weeks, days, or times of day? This is not a hypothesis-driven study; rather, by investigating the effects of time on the acoustic environments that listeners experience, we aim to get a sense of how long sampling periods should be, how many are required, and across what time spacing they should be collected to obtain reliable and valid data about listeners' acoustic environments.

## III. METHODS

### A. Participants

Data for the present study came from Parker *et al.* (2022) and extends the preliminary analyses in Tufts *et al.* (2020). This study used a sample of 34 college students (18–24 years old, mean = 20.26 years old, and 23 females) for whom three separate week-long runs of dosimeter data were available. All participants had normal middle ear function based on tympanometry and otoscopy, normal hearing based on audiometric thresholds of ≤20 dB hearing level (HL) for octave and semi-octave frequencies from 125 to 8000 Hz (ANSI, 2004), and normal or near-normal speech-in-noise perception based on the QuickSIN (Killion *et al.*, 2004). The study was approved by the Institutional Review Board at the University of Connecticut (IRB H14-214; approval date, 9/11/2018), and all participants received compensation for participation. Data were collected during the fall, winter, and spring of the 2018–2019 academic year. Data were not collected during the university recesses (e.g., fall, winter, or spring break).

### B. Dosimetry

Each participant was given an Etymotic Research (ER-200DW8, Elk Grove Village, IL) personal noise dosimeter and trained on its use. Dosimeter assignment was pseudorandomized; the dosimeter given to a participant was generally not the same from run to run but could have been by chance. Participants were instructed to wear the dosimeter on their clothing close to their ears and not cover the microphone. Participants were instructed to not wear the dosimeter during sleeping, showering, or physical activities that would risk damage to the device but to keep the device nearby (e.g., on a nightstand or countertop). Thus, the dosimeter was collecting data at all hours, including during sleep. The dosimeters sampled the environment at a rate of 4.54 Hz and calculated the resulting long-term average equivalent *A*-weighted level in dB (LAeq) over successive 3.75-min windows, giving 16 LAeqs per hour.

Dosimeters have the advantage of being simple to use and are designed for the purpose of measuring sound levels. However, because they are primarily concerned with noise dose measurements, they do not typically record continuous values for low sound levels, instead quantifying levels below a specified threshold as zero. Dosimeters in this study were configured with a 70-dBA threshold such that sound pressure levels < 70 dBA were essentially recorded as 0 dBA. During a given 3.75-min window, if the threshold was never exceeded, the dosimeter returned a value of 0 dB for that window's LAeq. If the threshold was exceeded for part or all of a window, a nonzero LAeq was returned. Given the sampling rate of the dosimeter, the minimum possible nonzero LAeq was 40 dB.

We treat each of these 3.75-min windows as individual *samples* of the participant's acoustic environment rather than integrating measurements across longer time periods, as is typical when reporting dosimetric data. By treating each short-term LAeq as an individual sample, we are able to use linear mixed models with random effects for participants to estimate the fixed effects of time scale on sound levels (e.g., Jorgensen *et al.*, 2023a). In this approach, however, using zero and nonzero samples in the analysis is problematic as it could result in misleading estimates of sound levels within each time scale, could obscure the impact of low sound level environments on time scale effects, and would complicate the interpretation of the analysis results. Thus, for most time scales, the present study estimated the effects of time scale on acoustic environment demand by treating samples < 40 dBA as a category and characterizing the proportions of lower (<40 dB LAeq) and higher (≥40 dB LAeq) dosimeter samples in a time period, as well as the mean levels for dosimeter samples ≥40 dB LAeq. Greater proportions of sound levels ≥ 40 dB LAeq and higher mean levels for samples ≥ 40 dB LAeq in a time period suggest more demanding acoustic environments within that time period. The diversity of acoustic environments was characterized by computing the entropy of sound levels from the distributions of dosimeter samples ≥ 40 dB LAeq. The dosimeter settings and range of levels collected in this study (40–120 dB LAeq) are roughly comparable to those of other studies using dosimetry to quantify acoustic environments (e.g., Flamme *et al.*, 2012; Wu and Bentler, 2012).

Dosimeters were subjected to regular electroacoustic checks to ensure that they remained functioning and within acceptable tolerances. This was performed by presenting a 1-kHz narrowband signal in an Audioscan Verifit test box (Dorchester, Ontario, Canada) containing the dosimeter and a separate microphone attached to a Larson-Davis 824 type 1 sound level meter (Depew, New York) located outside the test box. The dosimeters can return their measured sound level using the “QuickCheck” mode on the device. Dosimeters were considered within calibration if their mean checked level over three measurements was within 2.5 dB of the mean sound pressure level recorded by the sound level meter. The dosimeters were turned on as the participant left the laboratory, and the start time was recorded. The off button on the dosimeter was disabled, therefore, the dosimeter ran continuously until the participant returned to the laboratory a week later or there was a technical problem with the dosimeter. If there was a technical problem, the participant returned to the laboratory as soon as possible and was given a new device to finish the sampling run. Dosimeter data were downloaded from the device using the accompanying Etymotic Research Software (ER200D Utility Suite version 4.04). Timestamps were added using custom matlab functions (Natick MA). The three runs (weeks of data collection for each participant) were labeled *A*, *B*, and *C*. When the dosimeter malfunctioned and the participant had to swap out dosimeters mid-run, the two runs were sub-labeled *a* and *b* (e.g., *Aa*, *Ab*, etc.). Such runs were not included in all analyses when a break in the run would complicate answering the research question. When these runs were excluded from analyses is detailed in Sec. IV. Most participants completed the three sampling runs; two participants completed runs *A* and *B* but not run *C*. As a result of breaks in the academic calendar (fall, winter, and spring recess), participants did not complete the runs with equal time off in between. Details of these differences are described in the results in Sec. IV.

### C. Analyses

Differences in acoustic environment demand and diversity were characterized across four time scales such that

• season: The effect of season on acoustic environment demand and diversity was characterized by comparing runs completed in the fall, winter, and spring;
• week: The effect of week on acoustic environment demand and diversity was characterized by comparing the three week-long runs (*A*, *B*, and *C*);
• day of the week: The effect of day of the week on acoustic environment demand and diversity was characterized by collapsing the three runs and comparing days of the week to each other (Sunday, Monday, Tuesday, Wednesday, Thursday, Friday, and Saturday); and
• time of day: The effect of time of day on acoustic environment demand was characterized by determining how sound level changed as a function of the time of day, using the timestamp for dosimeter sample. Acoustic environment diversity was not evaluated for this timescale because its proxy measure, entropy, requires the entire distribution of values within the timescale and, thus, no meaningful comparisons could be made without arbitrarily discretizing the day into smaller time scales.

Acoustic environment demand and diversity were characterized using the LAeq values of the samples. From these, two metrics of demand and one metric of diversity were calculated:

• Acoustic environment demand was first measured by comparing the proportion of samples < 40 dB LAeq (the lowest nonzero LAeq) to the proportion of samples ≥ 40 dB LAeq. The reason for this analysis on the proportions is because windows where the LAeq was < 40 dB were recorded by the dosimeter as null values, hence, creating noncontinuous LAeq data. Although other studies have treated these null values as 0 dB and integrated across all levels (e.g., Flamme *et al.*, 2012), we took the approach of performing separate analyses on the proportions of samples ≥ 40 dB LAeq. Because the actual levels of sounds below the dosimeter threshold are unknown and each sample is treated as a discrete point, the null values represent a category (samples < 40 dB LAeq). Therefore, for most time scales, we performed a separate analysis for null values using proportions rather than treating LAeq as a continuous variable. From the proportions, a likelihood of samples being ≥40 dB LAeq was estimated. A higher proportion of samples ≥40 dB LAeq suggests greater demand in that sampling period;
• acoustic environment demand was also measured by analyzing only the samples ≥ 40 dB LAeq, treated continuously. Mean differences in LAeq values for samples ≥ 40 dB LAeq were analyzed across different time periods. Higher mean LAeq values suggest greater demand in that sampling period. Note that LAeq values were not combined across samples to yield equivalent levels for longer time periods as would be done if the goal were to characterize participants' noise exposures. Instead, samples are treated individually, and means across different time scales are estimated using mixed effects (detailed below); and
• acoustic environment diversity was quantified using the entropy of the LAeq levels for samples that were ≥ 40 dB LAeq (Jorgensen *et al.*, 2023b; Wu *et al.*, 2023). Recall that entropy quantifies diversity as a function of the predictability of some set of events or properties; less predictability results in a more uniform probability density function, which, in turn, results in a higher entropy value, indicating greater diversity. In the current study, higher entropy values indicate less predictable acoustic environments (i.e., more uniform probability density functions of the LAeq values in the environment) and, thus, greater acoustic environment diversity. To calculate the entropy, LAeq values were binned in 3 dB bins from the lowest (40 dB) to highest observed (120 dB) LAeq values. A 3-dB bin size was chosen as it resulted in a reasonably good fit to the distribution of the data, is theoretically meaningful as a doubling of sound intensity, is a common value in audiologic applications such as defining filter cut-offs, and represents a small but noticeable change in perceived loudness for real-world stimuli (e.g., Caswell-Midwinter and Whitmer, 2019; McShefferty *et al.* 2015). Then, the probability of each bin was multiplied by the base-2 log of the bin's probability. The products of the probability of each bin and the base-2 log of the bin's probability were then summed and multiplied by −1 to give the entropy value (Jorgensen *et al.*, 2023b; Shannon, 1948). Entropy values were calculated for each participant within each time scale except for time of day, as noted above. Note that entropy is dimensionless and can only be used to compare values within this study; entropy values are not absolute and not meaningful to compare across studies.

Generally, this study followed the statistical analysis guidelines described in Oleson *et al.* (2022). Those guidelines specifically described the use of linear mixed effect models in analyzing repeated measures from ecological momentary assessments collected from listeners' daily lives. This approach is appropriate for the dataset in the present study because like ecological momentary assessments, sound levels in the present study are nested within participants. Further, linear mixed models are generally robust to violations of distributional assumptions, which can arise in real-world data as a result of imbalances (Murphy *et al.*, 2022; Schielzeth *et al.*, 2020). Linear mixed models are particularly useful for the present study as the approach allows for flexible random effects structures that can allow participants to vary in their baseline differences in sound levels (intercepts) and their change in sound levels across time scales (slopes). For any given time scale, the dependent variable was proportions of samples ≥ 40 dB LAeq, mean levels for samples ≥ 40 dB LAeq, or LAeq entropy. The independent variable was the time scale grouping (season, week, day of the week, and time of day). Because we do not assume *a priori* that participants would have similar sound levels or similar changes in sound levels across time scales, participants were, when possible, given random intercepts and slopes for each mixed effects model. In some cases, only random intercepts were included due to non-convergence (specified in Sec. IV) or, in the case of entropy calculations, too few observations per participant to fit both random effects. For proportions of samples ≥ 40 dB LAeq, generalized linear mixed effects models with logit link functions were used. For mean LAeqs for samples ≥ 40 dB and entropy of sound levels, linear mixed effects models were used. To compute *p*-values, *Z*-tests with infinite degrees of freedom were used for generalized linear mixed effects models, and *t*-tests using the Satterthwaite method (Satterthwaite, 1946) to calculate degrees of freedom were employed for linear mixed effects models, and *a priori* pairwise comparisons were conducted where appropriate, depending on the research question. When conducted, *p*-values were adjusted using false discovery rate corrections (Glickman *et al.*, 2014). Model assumptions were evaluated by visual inspection of diagnostic plots (fitted vs residuals and quantile-quantile plots). No assumption violations were detected. Raw effect sizes, either in mean differences (for normal linear mixed models) or odds ratios (for logistic mixed models) are reported where applicable.

In addition to group-level analyses, individual differences were also investigated. For each time scale (i.e., season, week, day, and time of day), the differences in sample proportions, mean levels, and entropy within each individual are presented. To give a sense of the individual variance, the ranges of within-individual differences across the different time periods are plotted and described. Differences in the proportions of samples ≥ 40 dB LAeq, the mean levels for samples ≥ 40 dB LAeq, and LAeq entropy between each pair of time periods (pairs of seasons, weeks, and days) for each individual participant were computed and plotted. For within-individual differences in sound levels by time of day, individual regressions were fit for each participant and plotted. Further, intraclass correlation coefficients for the random effects are provided for each model, indicating the amount of the variance that can be attributed only to clustering within individuals. Large intraclass correlation coefficients would indicate that most of the variance is accounted for just by the random effects for individual participants. The SDs for random intercepts and slopes are provided. The marginal *R*^2^ values of the models are also given, which estimate the amount of variance in the model accounted for by the fixed effects. That is, large marginal *R*^2^ values would indicate that most of the variance is accounted for by the time scale effect, whereas small values would indicate little of the variance is accounted for by the time scale effect. Pearson correlations are also reported between the entropy and mean sound levels within each time scale for samples ≥ 40 dB LAeq to determine the extent to which mean sound levels were independent of sound level entropy. The statistical results are described in Sec. IV. For detailed model output for significant results, please see the supplementary material. All analyses were performed using *R* (version 4.3.1, Beagle Scouts; R Core Team, 2023) and the lme4 (Bates *et al.*, 2015) and emmeans (Lenth, 2021) packages.

## IV. RESULTS

**FIG. 1.**
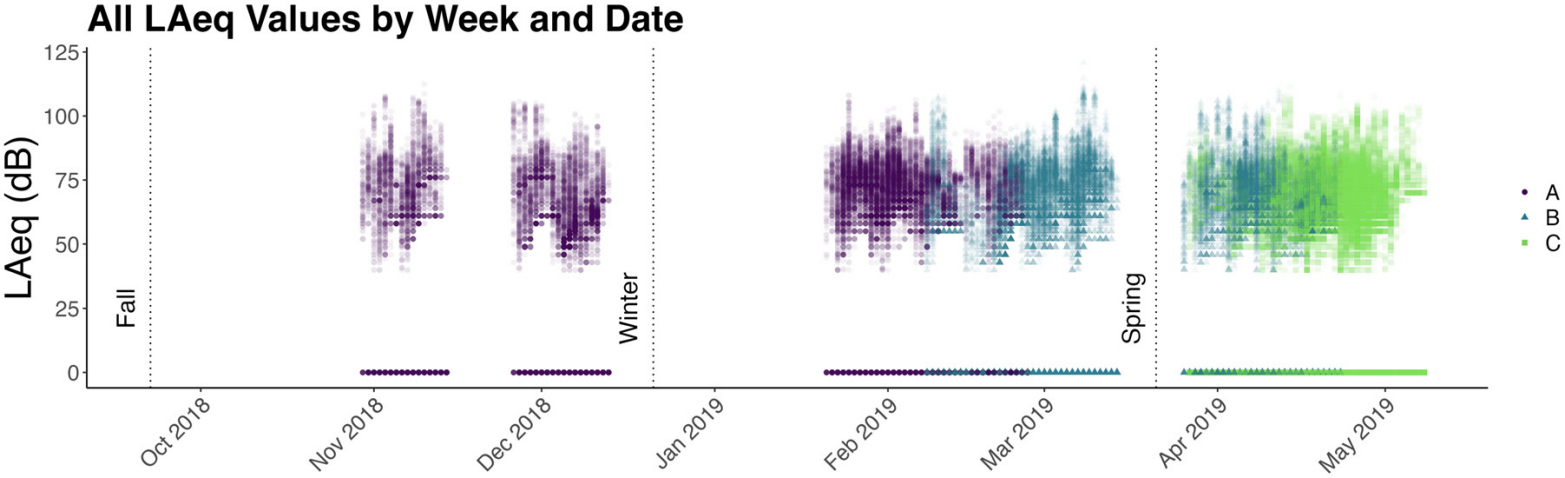
Individual LAeq samples as a function of individual days. The vertical spread on the *y* axis for a given point on the *x* axis indicates the range of sound levels recorded for all participants wearing dosimeters on a given day. Runs (full runs only) are distinguished by symbol and color. Dots along the zero line represent samples during which the dosimeter threshold was never exceeded.

The full dataset comprised 267 520 LAeq samples across 34 participants. To investigate differences between weeks and between seasons, the only runs included in the analysis were those in which the dosimeter did not have to be switched out during the week because of malfunction (Fig. 1). That is, the data included in season- and week-level analyses only included runs in which continuous data across the week were available. These complete runs included 29 participants for run *A*, 31 for run *B*, and 31 for run *C*, with a total of 238 095 LAeq samples (77 581 for run *A*, 78 260 for run *B*, and 82 254 for run *C*). The mean time spacing between runs *A* and *B* was 74 days (range, 7–153; SD = 36) and the mean time spacing between runs *B* and *C* was 32 days (range, 8–64; SD = 16).

### A. Acoustic environment demand and diversity between seasons

First, we investigated whether acoustic environment demand and/or diversity varied across different seasons. To do this, samples were divided by astronomical season based on the start and end season dates for 2018–2019. Of the total samples, 34 945 occurred in the fall (September 22–December 20), 82 443 occurred in the winter (December 21–March 19), and 120 627 occurred in the spring (March 21–June 20). Of the samples ≥ 40 dB LAeq, 11 275 occurred in the fall, 29 403 occurred in the winter, and 40 066 occurred in the spring. Note that the fall had fewer datapoints than the other seasons as most participants completed the runs in the winter and spring.

**FIG. 2.**
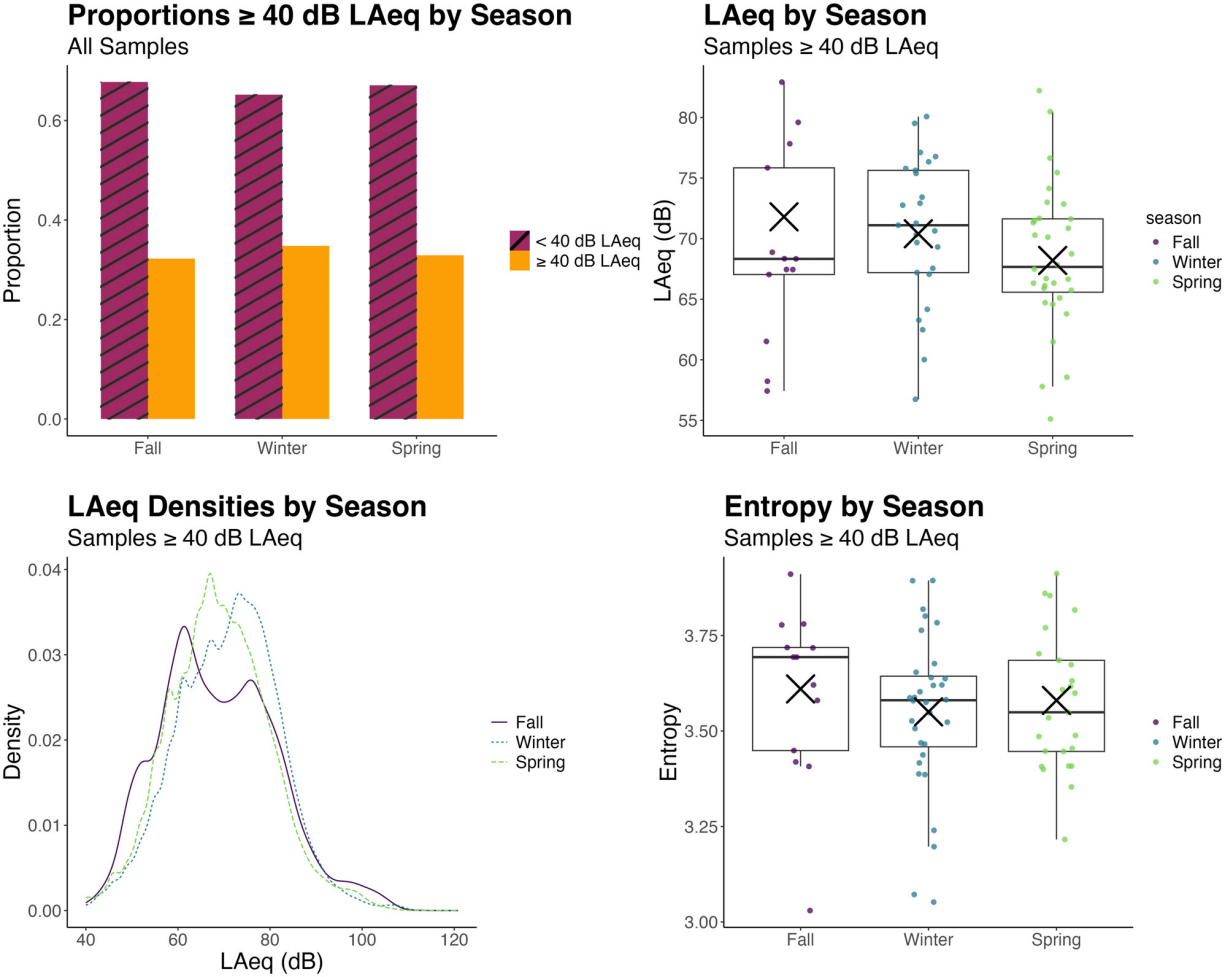
(Top left) Acoustic environment demand as proportions of samples ≥ 40 dB LAeq per season and (top right) acoustic environment demand as mean LAeqs for samples ≥ 40 dB LAeq for each season (shown averaged within subjects) are displayed. Dots are mean LAeq values for each subject. “×” symbols are model-estimated mean LAeq values per season. (Bottom left) Acoustic environment diversity as kernel density estimates of probability density functions for LAeqs for seasons and (bottom right) accompanying LAeq entropy values for each subject for each season are shown. Dots are entropy values for each subject. “×” symbols are model-estimated mean entropy values per season. Winter had the highest proportions of samples ≥40 dBA and fall had the highest LAeqs for samples ≥ 40 dB, indicating greater acoustic environment demand than in the spring. The distributions and entropy of LAeq values did not differ between seasons, suggesting no differences in acoustic environment diversity among seasons.

Differences in acoustic environment demand (proportions of samples ≥ 40 dB LAeq and mean levels for samples ≥ 40 dB LAeq) between seasons were small and no differences in acoustic environment diversity (LAeq entropy) were observed. Effects of season on acoustic environment demand are depicted as proportions ≥ 40 dB LAeq in the top left panel of Fig. 2 and as mean LAeq values for samples ≥ 40 dB LAeq in the top right panel. Note that the boxplots show the raw data using the standard quartiles and median line as well as an “×”symbol for the model-estimated means for the fixed effects of season. Most samples were < 40 dB LAeq; recall, however, that dosimeters ran continuously, including throughout the night. The winter had the highest proportion of samples ≥ 40 dB (0.35) and the fall had the lowest proportion of samples (0.32). For detailed model results, see the supplementary material, Table I. Pairwise comparisons showed the only significant difference among the seasons was between the fall and the winter, where samples in the winter were 1.27 times as likely to be ≥ 40 dB LAeq than those in the fall (*z*= –2.49, *p =* 0.038). For samples ≥ 40 dB LAeq, the fall had the highest sound levels with an average LAeq of 72 dB, and the spring had the lowest sound levels with an average LAeq of 68 dB (supplementary material, Table II). Pairwise comparisons showed seasonal differences were significant between the fall and spring (*z* = 3.14, *p =* 0.005) and the winter and spring (*z* = 2.34, *p =* 0.029) but not between the fall and winter. Kernel density estimates of probability density functions for LAeq for each season are shown in the bottom left panel of Fig. 2. A taller, more narrow probability density function indicates greater predictability and results in a lower entropy value. For example, the spring has a taller, more narrow probability density function than the fall and winter, and this is reflected in lower mean entropy values for the spring (Fig. 2, bottom right panel). However, differences in entropy between seasons were not significant [*F*(2) = 0.68, *p* = 0.512]. The correlation between LAeq entropy and mean LAeq for samples ≥ 40 dBA by season was not significant (*r* = 0.01, *p* = 0.93).

**FIG. 3.**
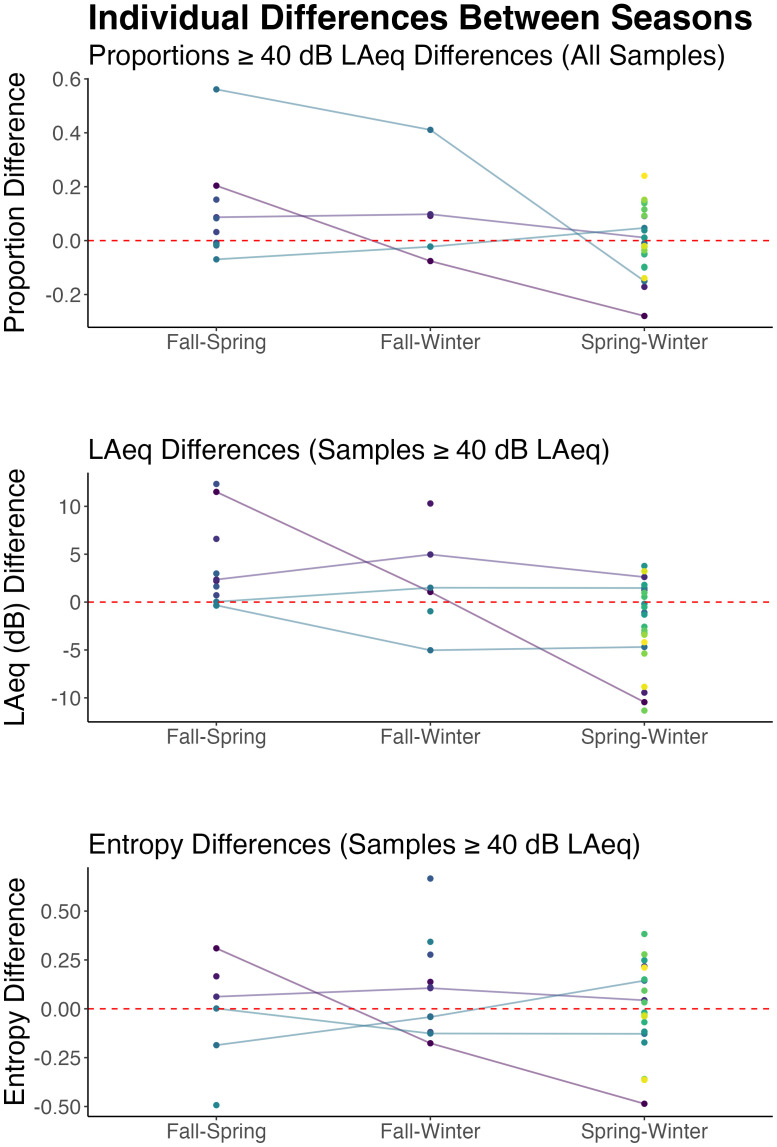
Within-individual differences between seasons for proportions≥ 40 dB LAeq (top), LAeq values (for samples ≥ 40 dB LAeq; middle), and entropy (bottom) are shown. Each line and dot color indicate a single participant. The dotted red line indicates no change across seasons. Not all participants have lines connecting dots across seasons as not all participants completed one run for each season. Some participants completed two runs in one season and one in another season, in which case, they only have one point for a pairwise comparison (all datapoints within a single season are averaged). Most lines/dots are near the no-change line with some clear exceptions, indicating some individuals did have large between-season differences.

Differences between seasons within individuals are shown in Fig. 3. The red dotted line indicates no change for a given pair of seasons; individual tracings or dots close to the dotted line indicate less change between seasons. Note that few participants had one run in each season, and most participants do not have tracings connecting all pairwise comparisons. Because most participants completed data collection in winter and spring, there are more datapoints for individual differences between those seasons than between fall and winter and fall and spring. Most participants showed little change between seasons with some exceptions. For example, for one participant, the proportion of samples≥ 40 dB LAeq in the fall differed from the proportions in the spring and winter by nearly 0.6 and 0.5, respectively. The smallest difference for an individual participant was 0.008 (fall–spring). Baseline differences in proportions of samples ≥ 40 dB LAeq varied considerably among participants: The SD of the intercepts was 0.5. The intraclass correlation coefficient for the random effects was 0.09 and the marginal *R*^2^ of the model was 0.002, suggesting that little of the variance was explained either by season or participant. For samples ≥ 40 dB LAeq, between-season, within-individual differences in level ranged from less than 1 dB (fall–winter) to 12 dB (fall–spring). Like proportions, participants varied in their individual average sound levels: The SD for intercepts was 7 dB and the average SD for slopes across seasons was 4 dB. The intraclass correlation coefficient for the random effect was 0.29 and the marginal *R*^2^ was 0.01, again, suggesting that little of the variance was explained either by individual differences or seasons. Within-individual, between-season differences in LAeq entropy (samples ≥ 40 dB) ranged from 0.002 (fall–spring) to 0.67 (fall–winter). The SD of the random intercept for participant was 0.11, the intraclass correlation coefficient was 0.32, and the marginal *R*^2^ of the model was 0.02.

### B. Acoustic environment demand and diversity between weeks

**FIG. 4.**
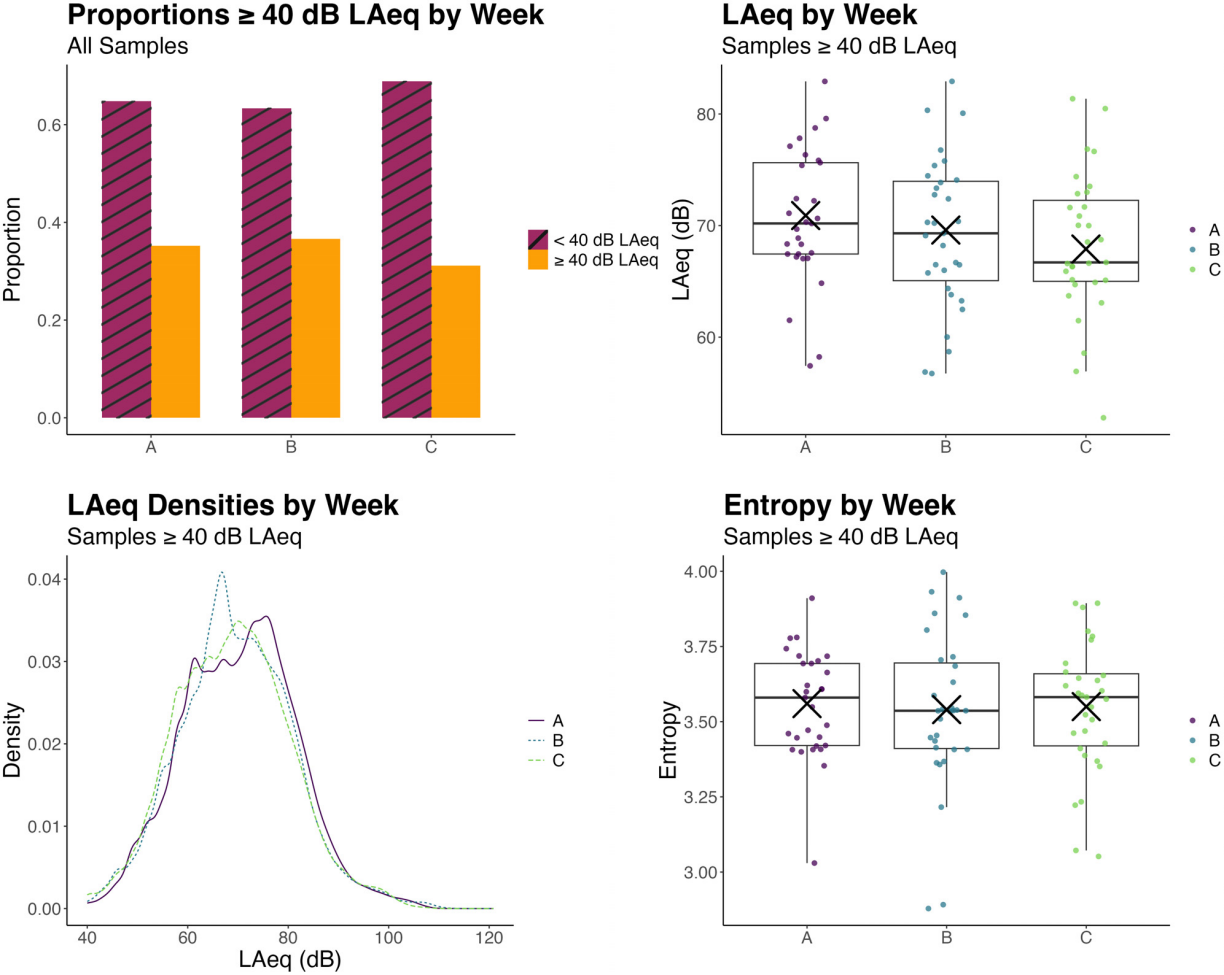
(Top left) Proportions of samples ≥ 40 dB LAeq per week (averaged within subjects) and (top right) boxplots of mean LAeq levels for samples ≥ 40 dB LAeq for each week (averaged within subjects) are shown. Dots represent mean LAeq values for each subject. “×” symbols represent model-estimated mean LAeq values for each week. (Bottom left) Kernel density estimates of probability density functions for LAeqs for full weeks and (bottom right) boxplots of the entropy values for each subject for each week are shown. Dots are the entropy values for each subject. “×” symbols represent model-estimated mean entropy values for each week. Week *B* had the largest proportion of samples ≥ 40 dB LAeq and week *A* had the highest sound levels for samples ≥ 40 dB LAeq, suggesting greater acoustic environment demand during weeks *A* and *B* than during week *C*. There were no differences in acoustic environment diversity (sound level entropy) between weeks.

Next, we investigated whether acoustic environment demand or diversity changed between weeks. Recall that participants completed three separate weeks (runs) of data collection with weeks *A* and *B* separated by, on average, approximately 2.5 months and weeks *B* and *C* separated by approximately 1 month. As with seasons, acoustic environment demand changed relatively little across weeks with no changes in diversity. Results for differences in acoustic environment demand and diversity across weeks are shown in Fig. 4. Proportions of samples ≥ 40 dB LAeq were 0.35 for week *A*, 0.37 for week *B*, and 0.31 for week *C* (supplementary material, Table III). Pairwise comparisons showed that the largest difference was between week *B* and week *C*, where samples in week *B* were 1.48 times as likely to be ≥ 40 dB LAeq than those in week *C* (*z* = 2.71, *p* = 0.021). Samples in week *A* were 1.25 times as likely to be ≥ 40 dB LAeq than samples in week *B* (*z* = 2.16, *p* = 0.047). Proportions of samples ≥ 40 dB LAeq did not differ between weeks *A* and *B* (*z*= –1.44, *p* = 0.149). Differences in average LAeq between weeks for samples ≥ 40 dB LAeq showed a similar pattern (supplementary material, Table IV). The grand mean LAeq for samples ≥ 40 dB LAeq was 70 dB. The largest difference was between weeks *A* and *C*, which differed by 3 dB (*z* = 3.61, *p* < 0.001). Weeks *B* and *C* differed by 2 dB (*z* = 2.24, *p* = 0.038). Weeks *A* and *B* did not differ significantly. There were no significant differences in LAeq entropy between weeks [*F*(2) = 0.09, *p* = 0.91], and the correlation between LAeq entropy and mean LAeq by week was not significant (*r*= –0.16, *p* = 0.12).

**FIG. 5.**
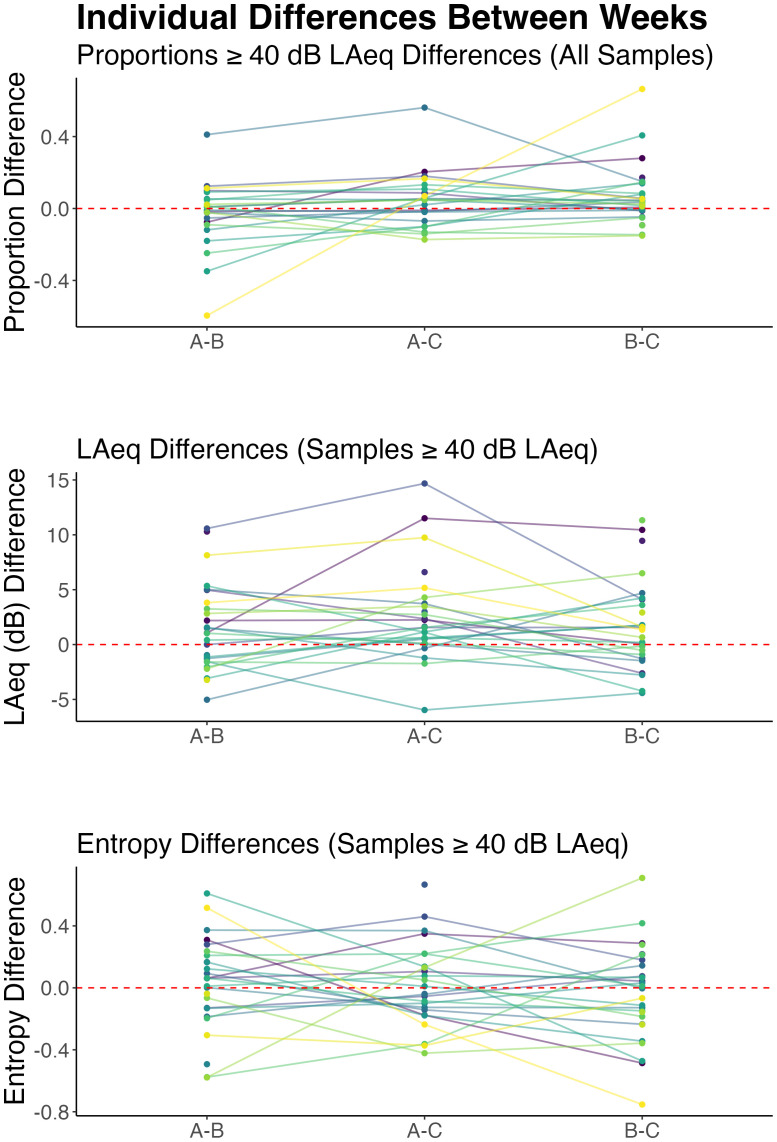
Within-individual differences between weeks for proportions ≥ 40 dB LAeq (top), mean LAeq values (for samples ≥ 40 dB LAeq; middle) and entropy (bottom). Each line and dot color indicate a single participant. The dotted red line indicates no change across weeks. Although the data are more concentrated around the no-change line, some participants show large changes in acoustic environment demand (top two panels) and diversity (bottom) across weeks.

Acoustic environment demand and diversity also changed little across weeks on the individual level. Differences between weeks for each individual participant are depicted in Fig. 5. Most participants showed changes around zero (no change), although some individuals showed more dramatic changes. For example, the most extreme participant showed large changes between weeks *A* and *B* and *A* and *C* but little change between *B* and *C*, suggesting that week *A* may have been an outlying week for that participant. For proportions of samples ≥ 40 dB LAeq, within-individual, between-week differences ranged from 0.004 to 0.66. The intraclass correlation coefficient for the random effects from the generalized linear mixed effects model for proportion differences was 0.18, suggesting that only a small portion of the variance was accounted for by individual differences. Although most participants did not show large changes in proportions, across weeks of samples ≥ 40 dB LAeq, they did differ considerably in their individual proportion baselines; participants varied in their intercepts by a SD of 0.48. The marginal *R*^2^ of the model was 0.006—almost none of the variance was accounted for by the fixed effect of week. Similar results were observed for the average LAeq of samples ≥ 40 dBA. For average LAeq values when the sample was ≥ 40 dB LAeq, participants varied in their intercepts by a SD of 6 dB and in their slopes (averaged between runs) by a SD of 4 dB. Between-week differences within individuals ranged from 0 dB to 15 dB. The intraclass correlation coefficient for the random effects was 0.31 and the marginal *R*^2^ of the model was 0.01; one-third of the variance was accounted for by individual differences but essentially none by the fixed effects. Within-individual, between-week differences in entropy ranged from 0.002 to 0.75. The SD of the intercept was 0.085, the intraclass correlation coefficient for the random intercept was 0.15, and the marginal *R*^2^ value was 0.002.

### C. Acoustic environment demand and diversity between days of the week

Next, we considered whether acoustic environment demand or diversity changed across the week—that is, whether demand and diversity changed from day to day. For this analysis, all samples were included in the dataset. That is, runs where the dosimeter malfunctioned and had to be switched out partway through the run were included as these data could still be meaningfully analyzed on the day level. This dataset then comprised 38 255 datapoints for Sunday, 37 444 datapoints for Monday, 38 729 datapoints for Tuesday, 37 877 datapoints for Wednesday, 37 795 datapoints for Thursday, 38 945 datapoints for Friday, and 38 475 datapoints for Saturday.

**FIG. 6.**
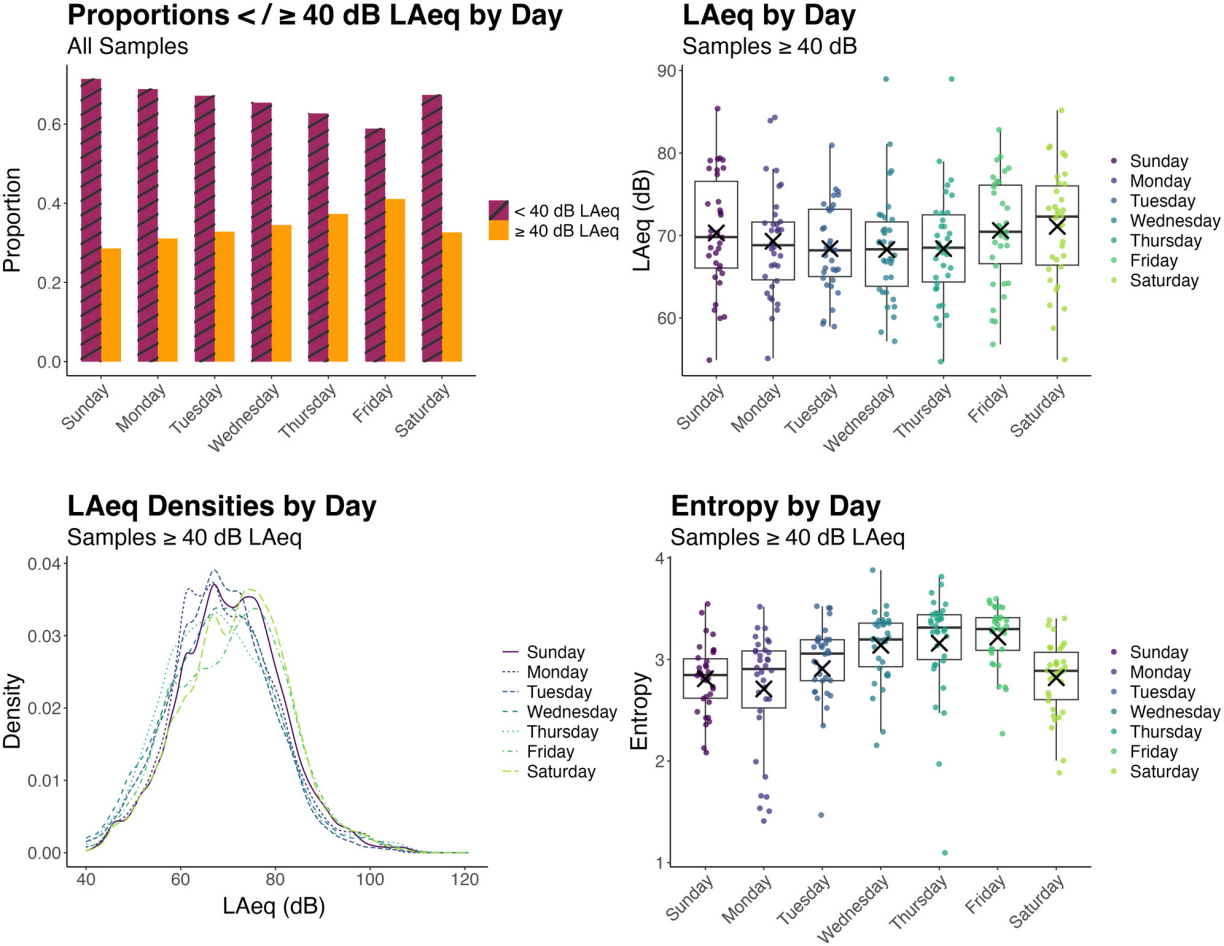
(Top left) Proportions of samples ≥ 40 dB LAeq per day.and (top right) boxplots of mean LAeq for samples ≥ 40 dB LAeq for each day (averaged within subjects across sampling runs) are shown. Dots represent mean LAeq values for each subject. “×” symbols represent model-estimated mean LAeq values for each day. (Bottom left) Kernel density estimates of probability density functions for mean LAeqs for days and (bottom right) boxplots of the entropy values for each subject for each day (averaged within subjects across sampling runs) are shown. Dots represent mean entropy values for subjects. “×” symbols represent model-estimated mean entropy values for each day. Acoustic environment demand was different on the weekends than on weekdays; proportions of samples ≥ 40 dB LAeq increased throughout the week from Sunday to Friday, dropping down again on Saturday. However, for samples ≥ 40 dB LAeq, mean levels were higher on the weekend than on weekdays. Weekdays showed greater environment diversity (higher entropy) than weekends.

The general takeaway from the day-of-the-week analysis is that differences in demand and diversity occurred across days of the week, particularly between weekdays and weekends: Weekends had fewer samples that were ≥ 40 dB LAeq but higher mean levels for those samples that were ≥ 40 dB LAeq, and weekdays were more diverse than weekends. Effects of day of the week on acoustic environment demand and diversity are shown in Fig. 6. Beginning with Sunday, acoustic environment demand based on proportions of samples ≥ 40 dB LAeq increased throughout the week, except for Saturday. Proportions of samples ≥ 40 dB LAeq increased nearly monotonically throughout the week with days ordered by proportion (smallest to largest) of samples ≥ 40 dB LAeq as: Sunday, Monday, Saturday, Tuesday, Wednesday, Thursday, Friday. Sunday had the lowest proportion of samples ≥ 40 dB LAeq (0.29) and differed significantly from all other days except Monday (supplementary material, Table V; all pairwise comparisons are provided in supplementary material, Table VI). Friday had the greatest proportion of samples ≥ 40 dB LAeq (0.41) and differed significantly from all other days. Odds ratios ranged from 0.55 (Sunday–Friday) to 1.52 (Friday–Saturday).

Although Saturday had a lower proportion of samples ≥ 40 dB LAeq than most other days, suggesting more time spent in environments with relatively low demand, it had the highest mean level for samples ≥ 40 dB LAeq, 71 dB, suggesting that the remainder of the time was spent in more demanding environments compared with other days of the week. Levels were lower on the weekdays, particularly on school nights (Sunday–Wednesday), than the weekends (Thursday–Saturday). The lowest sound levels were observed on Wednesday with a mean LAeq of 68 dB for samples ≥ 40 dB LAeq. Weekdays showed more similarity in level than weekends. Pairwise comparisons between days using false discovery rate corrections showed that the mean LAeq (for samples ≥ 40 dB LAeq) differed significantly between most pairs of days (supplementary material, Table VII, with all pairwise comparisons provided in supplementary material, Table VIII).

Acoustic environment diversity also increased from Monday to Friday, dropping back down on Saturday (supplementary material, Table IX). The low entropy observed on Saturday is consistent with the findings that Saturday had the highest mean LAeq but also the lowest proportion of samples ≥ 40 dB LAeq, suggesting Saturdays were characterized by greater contrasts in environmental levels compared with other days. The higher entropy values observed on Wednesday, Thursday, and Friday indicate that acoustic environments on these days were less predictable than those on Saturdays, Sundays, Mondays, and Tuesdays. Pairwise comparisons support this (supplementary material, Table X) with few significant differences observed between Wednesday and Friday and Saturday and Tuesday but significant differences between the Wednesday–Friday group and the Saturday–Tuesday group. LAeq entropy and mean LAeq by day were not correlated (*r* = 0.07, *p* = 0.30).

For most individuals, differences between days were small with larger differences observed between the weekdays and weekends (for plots of individual between-day differences, see supplementary material, Fig. 1). Like the results for seasons and weeks, there were a few individuals with clearly outlying days; in a given week, some participants had days with very demanding acoustic environments. Within-individual, between-day differences in proportions of samples ≥ 40 dB LAeq ranged from 0.0006 (Monday–Saturday) to 0.42 (Wednesday–Friday). Baseline variances in proportions were like those of the other time scale analyses with a standard deviation for the intercept of 0.52. The intraclass correlation coefficient for the random effects was 0.09 and the marginal *R*^2^ was 0.009; almost none of the variance was explained by the model. For samples ≥ 40 dB LAeq, within-individual, between-day differences in mean LAeq ranged from less than 1 dB (Tuesday–Wednesday) to 23 dB (Tuesday–Thursday). The SD for the intercept was 6 dB. The intraclass correlation coefficient for the random intercept was 0.25 and the marginal *R*^2^ of the model was 0.009. The smallest entropy difference within-individual between-days was 0.0001 (Sunday–Tuesday) and the largest entropy difference was 2.42 (Thursday–Friday). The SD of the intercept for entropy was 0.16, the intraclass correlation coefficient for the random intercept was 0.06, and the marginal *R*^2^ of the model was 0.08.

### D. Acoustic environment demand across time of day

**FIG. 7.**
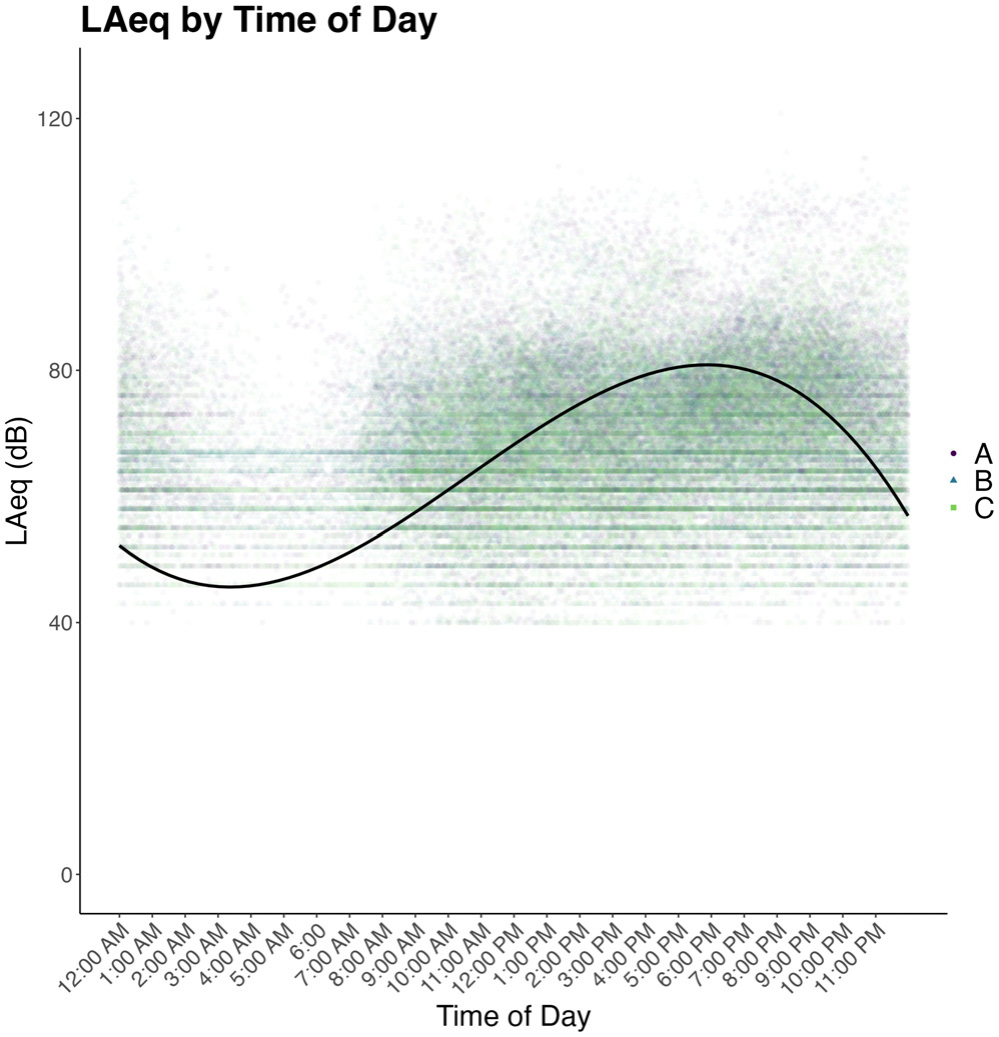
LAeq by time across the day. Dots are individual datapoints. Colors indicate the run (*A*, *B*, or *C*). The black function is a third-degree polynomial regression. The function indicates that sound levels are, on average, lowest in the early morning (approximately 3:00 AM) and highest in the early evening (approximately 6:00 PM). Sound levels increase from the morning until the early evening.

Finally, we investigated how sound levels changed across the day. As with the day-of-the-week analysis, all samples from all dosimeter runs were included in this analysis. LAeq increased from the early morning to the evening and then decreased again. To quantify this change, the timestamp of the dosimeter reading, with 1-min resolution, was treated as the independent variable. The dependent variable was the LAeq value at each minute timestamp, treated continuously and inclusive of samples with 0 dB LAeq. The change in LAeq by time of day with a fitted third-degree polynomial regression to account for the nonlinear shape of the change is shown in Fig. 7. Using the fitted function, times of day with the highest and lowest average LAeq values across all participants were identified. The time of day with the highest LAeq (inclusive of samples with 0 dB LAeq) was about 6:00 PM and the time of day with the lowest LAeq was about 3:30 AM. These timestamps then served as the bounds to create a two-piece linear regression, one for the daytime and one for nighttime. Only intercept for participant was included as a random effect because of model convergence. Time of day had a significant effect on LAeq, with LAeq increasing 0.05 dB for each minute from 3:22 AM to 5:53 PM and decreasing by 0.02 dB for each minute from 5:54 PM to 3:21 AM. These models are provided in the supplementary material in Tables XI and XII, respectively. The steepest slope of the function occurred between 11:00 PM and 12:00 AM, where the model-estimated LAeq dropped by 12.37 dB. The shallowest slope occurred between 5:00 and 6:00 PM, where the model-estimated LAeq increased by just 0.37 dB. Generally, the largest changes occurred throughout the late evening into the early morning (the total model-estimated drop in LAeq from 8:00 PM to 2:00 AM was 32 dB) and the mid-morning to mid-afternoon (the total model-estimated increase in LAeq from 9:00 AM to 3:00 PM was 20 dB). For the within-individual data, the peaks of the functions varied between approximately 4:00 and 10:00 PM, and the baseline level varied between participants during the day by a SD of 7 dB and during the night by 9 dB, but participants generally showed the same pattern: Sound levels peaked in the evening, decreased into the early morning, and then increased again throughout the day. The individual regressions are shown in the supplementary material, Fig. 2. For the daytime model, the intraclass correlation coefficient for the random intercept was 0.05 and the marginal *R*^2^ of the model was 0.144. For the nighttime model, the intraclass correlation coefficient for the random intercept was 0.08 and the marginal *R*^2^ of the model was 0.10. While still relatively low, these marginal *R*^2^ values are substantially higher than the values for other time scales, and the intraclass correlation coefficients are lower for most other time scales, suggesting that relative to the other time scales, the fixed effect of time across the day had a larger effect and explained more variance than the random effect of participant.

## V. DISCUSSION

The purpose of this study was to characterize how acoustic environment demand and diversity, quantified by sound levels, does or does not change across seasons, weeks, days of the week, and time of day. The primary motivation for characterizing changes in acoustic environment demand and diversity on different time scales was to inform the design of future studies of acoustic environments that are part of a person's daily life. If differences are known to occur for some or all time scales, choices such as the duration of sampling period (e.g., weeks, days, or hours), number of sampling periods (e.g., one vs multiple weeks, or sampling at different times of the day), and time of year of sampling (e.g., season) could affect the conclusions that are drawn. In this study, dosimeters were used to measure sound levels in the environments of a sample of college students with audiometrically normal hearing. Acoustic environment demand was quantified using proportions of samples ≥ 40 dB LAeq and mean levels for samples ≥ 40 dB LAeq (Christensen *et al.*, 2021; Wu and Bentler, 2012). The entropy of sound levels for samples ≥ 40 dB LAeq was used as an estimate of acoustic environment diversity (Christensen *et al.*, 2021; Gatehouse *et al.*, 2003; Wu and Bentler, 2012; Wu *et al.*, 2023; Jorgensen *et al.*, 2023b).

The first finding from this study was that sound levels were largely stable between the fall, winter, and spring. For proportion of samples ≥ 40 dB LAeq and mean sound levels for samples ≥ 40 dB LAeq, differences between seasons were small, with winter having a slightly higher likelihood of samples ≥ 40 dB LAeq than fall, and fall and winter having higher mean levels for samples ≥ 40 dB LAeq than spring. No differences in entropy were observed, suggesting that the predictability of sound levels does not vary as a function of season. The higher mean levels in fall and winter than spring could be the result of waxing and waning in sound levels due to seasonal activity patterns in the academic calendar. In the late spring as the semester winds down, students may spend more time in quiet study and less time in academic or social activities; this may be reflected in the lowest levels observed in the spring. In any case, the differences between seasons, even when significant, were small. The takeaway from these results is that by and large, the sampling season is unlikely to significantly bias measurement of acoustic environments.

The second finding from this study was that sound levels were stable across weeks when quantified as the proportion of sound levels ≥ 40 dB LAeq within a week and mean sound levels for samples ≥ 40 dB LAeq. Absolute proportion differences across all weeks were less than 4%. Mean sound levels (for samples with LAeq ≥ 40 dB) between weeks varied on the group level by less than 3 dB. This is not to say that there were not between-individual differences in baseline sound levels or changes in sound levels across weeks; recall from Fig. 5 that for samples ≥ 40 dB LAeq, participants differed in their mean sound levels within a week across a range of over 30 dB with a SD of about 6 dB and a slope deviation of about 4 dB. However, after accounting for these individual differences, the variance explained by the time period itself (week) was near zero. There were no differences in sound level entropy between weeks. The takeaway from these results is that, on average, a single week of sampling may be enough to reliably estimate the demand and diversity of a listener's typical acoustic environment.

The third finding from this study was that there were differences in acoustic environment demand and diversity across days of the week. Weekends (Saturday–Sunday) had more samples < 40 dB LAeq but higher mean sound levels when samples were ≥ 40 dB LAeq than weekdays. These findings are generally aligned with those of Chan *et al.* (2023) and the nonmusician population in Tufts and Skoe (2018). Weekdays had higher sound level entropy than weekends, indicating acoustic environments on weekdays were more diverse. Taken together, these results suggest that this population may spend more of the day on the weekends in quiet, but outside of those times, the sound levels are higher than on weekdays. The results also suggest that this population may experience more variable acoustic environments during the weekdays, perhaps as a result of a greater diversity of activities (class, extra-curriculars, etc.) during the week than on weekends. The absolute differences in proportions, levels, and entropy between days were, when significant, relatively small. However, the patterns of change across days of the week were clearer and more robust than those for weeks or seasons. These findings also offer support for high compliance in this study. Days of the week differences observed here are consistent with prior work and generally aligned with what is known about the lifestyles of American college students. The takeaway from these results is that day of the week is an important variable to consider when measuring acoustic environments.

The fourth finding from this study was that sound levels change systematically across the day. Averaged across days, the early hours of the morning showed the lowest sound levels, with sound levels increasing throughout the day until the early evening when they began to decrease again. These findings are aligned with those reported by Flamme *et al.* (2012), who similarly found that sound levels peak in the early evening and decrease until the early morning. We have extended those findings by using a balanced dataset, controlling for correlations among individual participants, and describing sound level changes across the day as a continuous function. Individual patterns of sound levels across the day were similar in their overall shape and peak times, but nonetheless there were differences in peaks across the day, with peak levels observed from the early afternoon to the late evening and trough levels observed across the morning. Because the dosimeter ran while participants were sleeping, it might be that waking and sleeping patterns exhibit heterogeneity among college-aged adults. The takeaway from these results is that time of day is an important variable to consider when measuring acoustic environments.

Despite generally small changes in acoustic environment demand and diversity across time scales on the group level, some individuals showed more extreme variation. This was particularly true on the longer time scales; the intraclass correlation coefficients decreased from weeks to days and became very small for levels across the day, suggesting that larger amounts of variance were captured by individual clustering differences on the longer time scales than on the short time scales. The marginal *R*^2^ values were near zero for most time scales, indicating that the fixed effect of time scale accounted for almost none of the variance in sound levels. Although the marginal *R*^2^ value was still relatively small for levels across the day, it was magnitudes larger than for the other time scales, whereas the effect size of the random effects shrunk to near zero. This suggests that for levels across the day, the time of day had a larger effect than the individual differences. Beyond the statistical measures from the models, the ranges of within-individual changes were smaller on shorter time scales, further supporting the idea that as the observation time window zooms out, individuals appear more different in their changes in auditory demand and diversity across time. More extreme outliers were observed on the season and week scales than on the day and time-of-day scales. The mixed effects approach is a powerful statistical method for measuring and accounting for these individual differences for studies collecting repeated, real-world data samples (e.g., Oleson *et al.*, 2022).

The findings of this study can be summarized as follows: Listeners' acoustic environments do change as a function of time scale to a varying extent. On the group level, larger changes are observed on smaller time scales, with smaller changes, on average, observed the more time is zoomed out. The most robust findings from this study are that day of the week and time of day seem to be more important in terms of having an effect on acoustic environment demand or diversity than the week or season. Given these findings, we offer the following suggestions with respect to the timing of sampling for research on listeners' acoustic environments:

• sampling periods should comprise at least 1 week;
• if the sampling period is longer than 1 week, weekend and weekdays should be balanced within and between participants;
• studies using sampling periods of only 2 or 3 days, particularly if they are not balanced between weekdays and weekends, could be biased. If measurements are only possible across a limited number of days, balance weekday and weekend days over the sampling period (e.g., Benítez-Barrera, 2023);
• complete 24-h days should be sampled when possible;
• participant-selected or random sampling at only a few time points during the day likely does not accurately represent the overall acoustic environments of listeners; and
• multiple sampling periods across months or seasons are probably not required for most purposes, but we encourage reporting on the months and seasons when dosimetry is conducted.

There are some important caveats to these findings. A limitation of this study is the missing continuous LAeq data between 0 and 40 dB due to the limitations of the dosimeter as described previously in Sec. III. Sound levels below the dosimeter threshold were treated essentially as silence because their true level is unknown. This limitation is not unique to this study as acoustic environments are frequently dichotomized along sound level parameters based on the limitations of the technology used to collect the data (e.g., Humes *et al.*, 2018), and dosimeters typically have a measurement threshold as they were designed primarily for noise dose estimation (e.g., Flamme *et al.*, 2012; Tufts and Skoe, 2018; Wu and Bentler, 2012). We argue that, while imperfect, these data can provide important evidence for how much acoustic environments change over time. This study does not aim to describe the exact sound pressure levels or details of the acoustic environments of these listeners on each time scale; rather, it aims to answer whether their acoustic environments differ from week to week, season to season, day to day, and across the day within a constrained 8-month period. These data allow us to answer those questions or at least provide insight into the answers. Dosimeters also have the advantage as they are purposely built to measure sound levels and able to be calibrated for that purpose of yielding accurate sound level measurements. Studies that use recorders to assess sound levels can estimate continuous values across the range of sound levels, but the transfer function of the recording device must be carefully measured and applied to the recordings as these devices do not natively provide accurate sound level readings (Smeds *et al.*, 2015; Wu *et al.*, 2018). Even when these transfer functions are applied, the limited input dynamic range of these recording devices can lead to quantization of higher-level inputs, causing errors in estimation of sound levels at higher levels (Benítez-Barrera *et al.*, 2023; Wu *et al.*, 2018). Even if the LAeq data from the dosimeters were continuous, however, we also recognize that LAeq values are only one way to quantify acoustic environments. Although sound levels are good indicators of the demand and diversity of an acoustic environment (Humes *et al.*, 2018; Jorgensen *et al.*, 2023b; Smeds *et al.*, 2015; Wu *et al.*, 2018), many other factors affect a listener's experience of how demanding or diverse an acoustic environment is, such as the types of signal and noise, the spatial orientation of signals and noise, visual cues, reverberation, listening activity, and situation importance (e.g., Jorgensen *et al.*, 2021), as well as listener factors such as hearing status and fatigue (e.g., Pichora-Fuller *et al.*, 2016). Future studies should investigate how these factors change over time in a listener's life and what the consequences not only for research methods but for audiologic intervention outcomes might be. New technologies will likely enable more granular data to be collected about the acoustic environment over even longer time scales with little intrusion into participants' lives.

One motivation for this study came from the fact that research on the acoustic environments of hearing aid and cochlear implant users typically use single, continuous sampling periods of highly varying lengths, collected generally without consideration for season or specific time of year. The acoustic environments of listeners who use hearing aids and cochlear implants are an important input for algorithm design, counseling, and technology choice, as well as an important outcome measurement. It is important to know, then, how time might affect the measurement of acoustic environments and how we interpret this body of work. On one hand, it is not possible to directly apply the findings from this study to studies on the acoustic environments of hearing aid users as the population in this study was college-aged students with normal hearing. The results of this study could suggest that the time of year or number of weeks sampled may have even smaller effects for other populations, particularly older adults who form the largest segment of the population with hearing loss. Prior work has shown that younger listeners have more demanding and diverse acoustic environments than older listeners (Jorgensen *et al.*, 2023a; Jorgensen *et al.*, 2023b; Wu and Bentler, 2012). Thus, it seems likely that older demographics may experience even less variance in acoustic environments over time. Of course, many demographic and lifestyle factors might affect the acoustic environments listeners experience in daily life (Ramírez-Esparza *et al.*, 2024; Benítez-Barrera *et al.*, 2023). Thus, caution is warranted when extrapolating findings from this study to other groups.

These data were collected within a single 8-month period. Longer time courses reveal more drastic differences in acoustic environments over time. Further, historical events affect acoustic environments in ways that were not necessarily reflected in this study. For example, the COVID-19 pandemic drastically changed acoustic environments, quieting urban environments and causing listeners to spend more time in quiet (Dunn, *et al.*, 2021; Lenzi *et al.*, 2021). It is also known that acoustic environments vary considerably over longer historical epochs with changes in the urban and rural landscape, technologies, and the natural ecosystem (e.g., Francomano *et al.*, 2021). Thus, studies of acoustic environments should also consider the temporal, historical, social-cultural, and political context within which the data were collected. Our hope is that the analytical framework developed here can facilitate addressing such questions.

Finally, we have described how acoustic environments change—or, often, do not change—across different time scales. We have not said anything directly about how *soundscapes* change. That is, our results do not necessarily provide insight into how perceptions of the acoustic environment might change over time or how contextual factors could interact with time in ways that lead to differences in how the acoustic environment is experienced by listeners at different time points. For example, even though season may not seem to have a large effect on the sound levels listeners experience, how they experience the acoustic environment, due to changes in listening activities, sociocultural contexts, or the types of sounds and their sources, may still very well change across seasons. The acoustic environment seems to change predictably across the week and across the day, but how these changes are perceived and how they are affected by various other listener and environmental factors remains to be systematically investigated.

## VI. CONCLUSION

This study investigated whether acoustic environment demand (proportions of sound levels ≥ 40 dB LAeq and mean sound levels for samples ≥ 40 dB LAeq) and diversity (entropy of sound levels ≥ 40 dB LAeq) changed across seasons, weeks, days, and across the day among a group of college-aged adults with normal hearing. On the group level, larger changes were observed on smaller time scales (days of the week and across the day) than between weeks or seasons. Individual differences were smaller when looking at days of the week and across the day than between weeks and seasons. These results suggest that a 1-week sampling period is likely sufficient to represent the typical acoustic environments of most listeners, at least within a relatively confined period such as a year. However, days of the week and time of day when data are collected should be considered and may affect the results, especially if the days of the week and times of day are not balanced across participants. Some individuals showed much greater differences (i.e., greater change) in acoustic environment demand and diversity than other participants, especially on longer time scales, suggesting that sample size and recruitment methods should be carefully considered when designing and interpreting the results from studies of real-world acoustic environments. Interpretation of findings from the current study should be tempered by the limitations of how the sound levels were sampled, the fact that the results of this study were obtained from a homogenous population and may not reflect acoustic environment differences across time in other demographic groups, and the fact that how the acoustic environment was perceived by listeners vis-à-vis the soundscape was not directly investigated.

## SUPPLEMENTARY MATERIAL

See the supplementary material for additional figures and tables for all statistical model results.

## Data Availability

The data that support the findings of this study are openly available in https://github.com/SoundscapeLab, and data are available on Open Science Framework https://osf.io/tfvjc/ (Skoe, 2025).
